# A Prospective Study on Correlation Between Postural Control Ability and Patient-Reported Outcome Measure After Total Ankle Arthroplasty

**DOI:** 10.3390/jcm15124499

**Published:** 2026-06-10

**Authors:** Sung Jae Kim, Sung-Hoo Kim, Seo-Won Kang, Byung-Ki Cho

**Affiliations:** 1 Department of Orthopaedic Surgery, Hanyang University Guri Hospital, Guri 11923, Republic of Korea; sung1383@hanmail.net; 2 Department of Orthopaedic Surgery, Chungbuk National University Hospital, Cheongju 28644, Republic of Korea; hoo414414@hanmail.net (S.-H.K.); arctic12345@gmail.com (S.-W.K.); 3 Department of Orthopaedic Surgery, College of Medicine, Chungbuk National University, Cheongju 28644, Republic of Korea

**Keywords:** ankle, arthroplasty, patient-reported outcome measure, postural control ability, posturography

## Abstract

**Background/Objectives**: Information on the effects of total ankle arthroplasty (TAA) on postural control ability and their clinical significance remains insufficient. This study aimed to investigate changes in postural control ability following TAA for end-stage ankle arthritis and to identify their correlation with clinical outcome measures. **Methods**: The study included 32 patients under the age of 70 who underwent TAA for end-stage osteoarthritis and were followed for at least 3 years. Clinical outcomes, including abilities in daily living and sports activities, were evaluated using the Foot and Ankle Outcome Score (FAOS) and the Foot and Ankle Ability Measure (FAAM). Postural control ability was assessed using the static and dynamic stability test modes of Biodex posturography. Spearman’s correlation coefficient (r) was analyzed to determine the linear association between postural control ability and clinical outcome measures. **Results**: The FAOS and FAAM scores significantly improved from a preoperative mean of 32.5 and 34.1 to 82.3 and 83.6 at the final follow-up (*p* < 0.001), respectively. Posturographic evaluation showed a mean overall stability index of 1.52 for static postural control and 2.56 for dynamic postural control at the final follow-up. FAOS demonstrated no significant correlation with static postural control (r = −0.11, *p* = 0.425), but showed a significant correlation with dynamic postural control (r = −0.35, *p* = 0.021). Similarly, FAAM scores did not correlate significantly with static postural control (r = −0.26, *p* = 0.103), but demonstrated a significant correlation with dynamic postural control (r = −0.57, *p* < 0.001). **Conclusions**: TAA failed to bring a statistically significant improvement in postural control ability. Dynamic postural control ability after TAA was found to have a significant correlation with patient-reported clinical outcomes. To enhance postoperative clinical outcomes, including abilities for daily living and sports activities, biomechanical assessment and targeted rehabilitation strategies to improve postural control ability may be helpful.

## 1. Introduction

Total ankle arthroplasty (TAA), a representative surgical procedure for the treatment of end-stage ankle arthritis, is known to preserve ankle joint mobility and anatomic alignment, thereby enabling superior gait performance and functional recovery [1,2,3,4,5,6,7]. However, ligament unbalancing and malalignment of prosthesis alter biomechanics of the ankle joint and can be risk factors for polyethylene wear, subluxation, osteolysis, gutter impingement, aseptic loosening, and early failure after TAA [8,9,10]. To restore a coronal plane alignment and plantigrade foot, an appropriate balancing of the medial and lateral ligamentous complex including the syndesmotic ligaments is critical. However, this procedure may result in sacrifice of physiological mechanoreceptors in the ligamentous complex around the ankle joint [11].

Because the design rationale of recent third-generation TAA includes minimal bone resection, retaining ligamentous support, and anatomic balancing [12], proprioception and postural control ability following the TAA may be preserved in comparison to old TAA systems. Postural balance is a complex function resulting from various neuromuscular processes and is modulated by somatosensory input, reorganization processing in the central nervous system, and neuromuscular responses [13]. The somatosensory input is known to consist of visual, vestibular, and proprioceptive information. Because most TAAs are suggested for elderly patients, postural control ability to maintain a stable balance in activities for daily living should be considered one of the critical factors for successful TAA [14]. However, studies on changes in postural control and proprioceptive abilities following TAA in end-stage ankle arthritis patients and their clinical significance remain insufficient [11,15].

Improvements in physical performance including activities of daily living may be influenced by several variables apart from patient motivation (intention to rehabilitation), such as residual pain or swelling, muscle imbalance, insufficient postural control, or deficits in proprioception [16,17]. Inappropriate balance control and postural sway in elderly patients may cause poor physical functioning, fear of falling, and activity limitation [11]. There are still debates regarding how postural control ability changes after TAA, and whether this ability can be improved by proprioceptive-oriented rehabilitation. Postural control ability following the TAA may have significant effects on the clinical outcomes, long-term survival rate of prosthesis, and return to sports activities. A notable insufficiency seems to exist in published studies regarding differences in postural control ability between normal, degenerative, and replaced ankles of elderly people.

This study aimed to investigate changes in postural control ability following TAA for end-stage ankle arthritis and to identify their correlation with clinical outcome measures. We hypothesized that TAA would bring an improvement in postural control ability, and this would positively affect the clinical outcomes including daily living and sport activities.

## 2. Materials and Methods

### 2.1. Study Subjects

**Figure 1:**
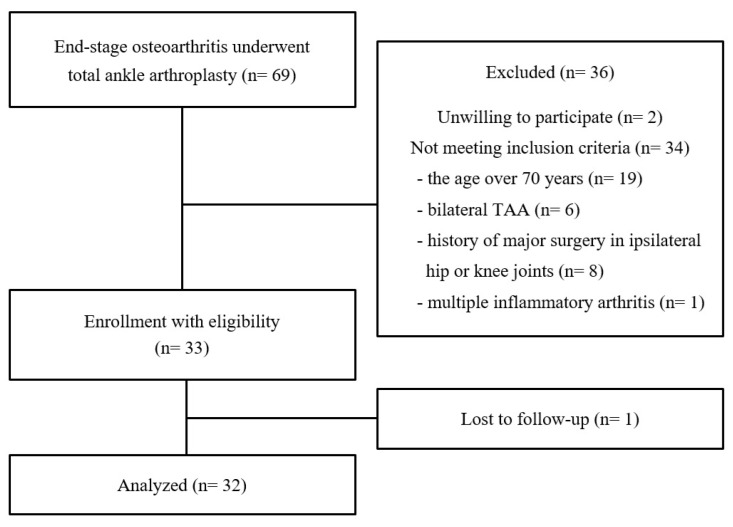
Consort flowchart of the current study.

This study was designed as a prospective clinical trial in a single institute. All the data were collected prospectively and analyzed retrospectively. All preoperative data were collected within 2 months prior to surgery. Postoperative clinical score questionnaires, radiological assessment, and posturographic evaluation were performed at 6 months, 1 year, and annually. This study included the patients with a follow-up of at least 3 years. Between January 2020 and June 2022, 65 patients (69 ankles) to be scheduled for the TAA for end-stage osteoarthritis were consecutively enrolled. Of these patients, 32 patients (32 ankles) eligible for inclusion and exclusion criteria were analysed in the current study (Figure 1).

**Figure 2:**
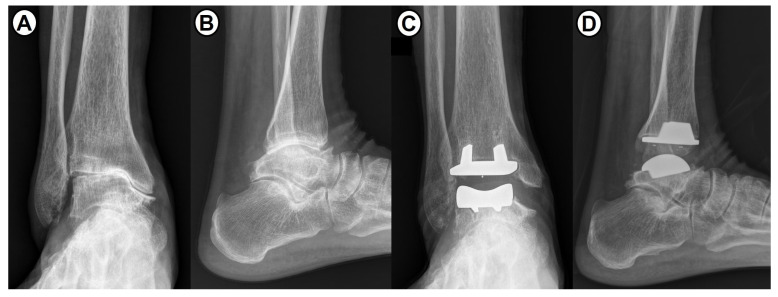
(**A**,**B**) Preoperative and (**C**,**D**) postoperative radiographs in a 64-year-old male with total ankle arthroplasty for severe osteoarthritis.

The inclusion criteria were as follows: (1) patients under the age of 70, (2) patients with unilateral TAA, (3) patients with follow-up for at least 3 years, (4) patients with no previous ankle ligament or fracture surgery history, (5) patients with no multiple inflammatory arthritis including rheumatoid arthritis, (6) patients with no history of major surgery in ipsilateral hip or knee joints, and (7) patients with no visual, vestibular, or neuromuscular impairments. Patients with prior septic arthritis or osteomyelitis around the ankle, avascular necrosis of the talus, neuropathic arthritis, peripheral vascular deficiency requiring angioplasty, coronal-plane deformities > 15 degrees, and body mass index (BMI) > 27 kg/m^2^ were excluded. All surgeries were performed by a single surgeon using 3-component mobile-bearing prosthesis (TARIC^®^, Implantcast GmbH, Buxtehude, Germany) (Figure 2).

At 4 weeks postoperatively, cast immobilization was removed and range of motion exercise was encouraged for all patients. From 6 weeks postoperatively, muscle strengthening exercises of the ankle and balance control training instructed by physical therapists twice a week were recommended for at least 3 months. Muscle strengthening exercises targeted the muscles responsible for dorsiflexion–plantarflexion and inversion–eversion of the ankle joint (tibialis anterior, triceps surae, tibialis posterior, and peroneal muscles). Physical therapists recommended isometric and isotonic strengthening exercises using a theraband or ball, and patients continued training at home as often as possible. Balance control training consisted of static tandem standing, one-leg standing on an unstable surface, and dynamic balance exercise (one-leg standing with ball toss). This rehabilitation program was randomly performed by three physical therapists. There was no specific rehabilitation program provided during the preoperative period. The study protocol and investigation were approved by the medical research ethics committee of the Institutional Review Board of Chungbuk National University Hospital (approval No. CBNUH 2020-01-002).

The patients had a mean age of 63.2 years (range, 57 to 69 years) at the time of surgery, with a mean follow-up duration of 51.2 months (range, 38 to 65 months) (Table 1). The current study included 14 male and 18 female patients. There were 19 affected right ankles and 13 affected left ankles. Patients had a mean body mass index (BMI) of 25.6 kg/m^2^, and 11 patients had osteoporotic bone mineral density values (T-score < −2.5) at the spine or hip. All patients exhibited mild to moderate varus or valgus malalignment less than 15 degrees.

### 2.2. Evaluation of Patient-Reported Clinical Outcomes

The clinical outcomes before and after surgery were assessed at regular intervals using the Foot and Ankle Outcome Score (FAOS) [18] and the Foot and Ankle Ability Measure (FAAM) [19]. The FAOS encompasses five subscales covering pain, symptoms, activities of daily living, sports and recreational function, and foot- and ankle-related quality of life. The FAAM comprises two subscales assessing activities of daily living and sports function. Each subscale was scored separately, and the results were converted to a 0–100 scale, where 100 points represented the best possible outcome with no symptoms or functional limitations, and 0 indicated the most severe symptoms and disability.

### 2.3. Evaluation of Radiographic Parameters

Two orthopedic surgeons independently evaluated the radiographs without access to clinical outcome data. These assessments included the deformities of the tibiotalar joint, the alignment of the implant, the progression of degenerative arthritis in adjacent joints, implant loosening or subsidence, periprosthetic osteolysis, and the presence of heterotopic ossification. All measurements were performed twice, 4 weeks apart, and the mean values were used for analysis.

### 2.4. Evaluation of Postural Control Ability

**Figure 3:**
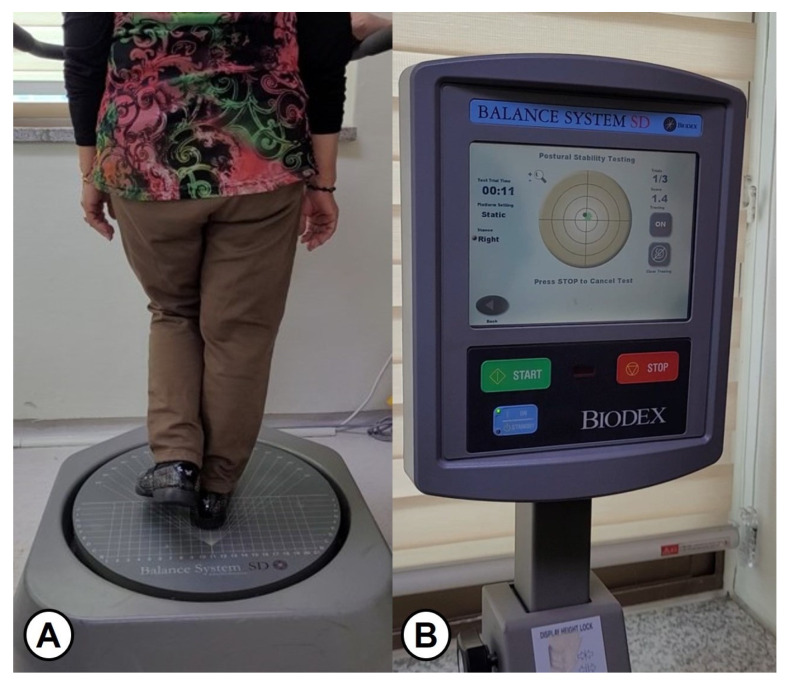
(**A**,**B**) Photographs demonstrating static and dynamic postural control evaluation using the Biodex posturography system, with continuous real-time tracking of center-of-gravity displacement shown on the monitor.

Static and dynamic postural control were assessed at regular intervals using Biodex posturography (Biodex Medical Systems, Shirley, NY, USA), which was verified to be reliable for evaluating postural balance [20]. During testing, subjects received visual biofeedback from a monitor displaying real-time shifts in their center of gravity (Figure 3). They were instructed to maintain a single-leg stance with eyes open while the platform automatically tilted and rotated, ranging from level 1 (most stable) to level 4 (less stable), for 20 s. Although the dynamic stability test using Biodex posturography is usually performed on a mobile platform (level 8 indicating the most unstable condition), a large number of patients could not complete the test at higher than level 5 during a pilot study in our institute. Taking into account the elderly patients with TAA, the dynamic stability test in this study was processed on a mobile platform at level 4. The balance platform was capable of tilting up to 20° in any direction within a 360° arc of motion. Under the supervision of the same examiner, each static stability test (on fixed platform) and dynamic stability test (on mobile platform) was performed twice with a 3 min rest interval, and the average values were recorded. Postural stability was quantified using the anterior–posterior stability index (APSI), medial–lateral stability index (MLSI), and overall stability index (OSI). These indices represent the horizontal deviations from the center of pressure (COP), with higher scores indicating greater displacement from the subject’s center of gravity and therefore poorer balance. To minimize learning effects, only one practice trial was conducted before the formal assessment.

### 2.5. Statistical Analysis

A power analysis for proper sample size calculation indicated that 13 patients per group would provide 80% statistical power for the comparison of the Overall Stability Index (OSI). A 95% confidence interval with a two-sided α level of 0.05 was applied to assess whether the between-group difference in OSI at final follow-up remained within the predefined noninferiority margin. The noninferiority margin was set at 0.8 OSI units, and an anticipated dropout rate of 5% was assumed based on previous studies. Accordingly, a minimum sample size of 13–14 patients per group was required to achieve adequate statistical power.

All collected data were analyzed using SPSS software (version 22.0; SPSS Inc., Chicago, IL, USA). Statistical significance was determined using a significance level of 0.05 and 95% confidence intervals. The assumption of normality was tested using both the Shapiro–Wilk and Kolmogorov–Smirnov methods. Comparisons of patient-reported clinical outcomes and postural control ability between the preoperative and postoperative in the same individuals were conducted using the Wilcoxon signed-rank test. The relationship between postural control ability and clinical outcome measures was evaluated using Spearman’s correlation coefficient (r). Correlation strength was interpreted based on the magnitude of significant correlation coefficients as strong (r = 0.7–1.0), moderate (r = 0.5–0.7), or weak (r = 0.3–0.5).

## 3. Results

### 3.1. The Changes of Patient-Reported Clinical Outcomes/Complications

The FAOS significantly improved from a preoperative mean of 32.5 to 82.3 points at the final follow-up (*p* < 0.001) (Table 2). Based on the analysis of each subscale, there were statistically significant improvements in all subscales. The mean FAAM score increased significantly from 34.1 preoperatively to 83.6 at final follow-up (*p* < 0.001). Significant improvements were observed in both daily and sport activity subscales.

As postoperative complications, there were two patients (6.25%) with marginal skin necrosis, one patient with superficial wound infection, one patient with deep peroneal nerve injury, and one patient with persistent medial ankle pain during gait by medial soft tissue and spur impingement. All marginal skin necrosis and superficial wound infection resolved following conservative treatment consisting of wound care and antibiotics, with no need for reoperation. There were no patients with revision surgeries requiring the change of prosthetic components.

### 3.2. The Changes in Radiographic Parameters

The mean tibiotalar angle improved from 8.1° of varus deformity preoperatively (range, 0 to 15°) to 1.6° of varus deformity at final follow-up (range, 0 to 5°). We reported one patient (3.1%) with malalignment of the tibial component (an angle greater than 10° between the articular surface of the tibial component and the anatomical axis of the tibia), one patient with subsidence of the talar component (a migration over 5 mm), one patient with periprosthetic osteolysis (a radiolucent line > 2 mm), and one patient with heterotopic ossification. Malalignment of the talar component, loosening of the tibial component (a change in alignment exceeding 2°), progressive arthritis in the adjacent joint, and subluxation of polyethylene insert were not found.

### 3.3. The Changes in Static and Dynamic Postural Control Ability

Static postural control ability (overall stability index) was changed from preoperative mean 1.63 to 1.52 at the final follow-up (*p* = 0.551) (Table 3). Dynamic postural control ability (overall stability index) was changed from a preoperative mean of 2.74 to 2.56 at the final follow-up (*p* = 0.348) (Table 4). Although both static and dynamic postural control abilities showed a trend toward lower stability index after surgery, no statistically significant differences were found when compared to preoperative evaluation.

### 3.4. Correlation Between Postural Control Ability and Clinical Outcome Measures

FAOS demonstrated no significant correlation with static postural control (r = −0.11, *p* = 0.425), but showed a low linear association with dynamic postural control (r = −0.35, *p* = 0.021) (Table 5). Similarly, FAAM scores did not correlate significantly with static postural control (r = −0.26, *p* = 0.103), but demonstrated a moderate linear association with dynamic postural control (r = −0.57, *p* < 0.001).

## 4. Discussion

This prospective study reports the changes in postural control ability following TAA for end-stage ankle arthritis and their clinical significance. The most important finding was that TAA failed to bring a statistically significant improvement in postural control ability. In addition, dynamic postural control ability after TAA had a significant correlation with patient-reported clinical outcomes. This information may be helpful to an advanced rehabilitation strategy to improve postoperative clinical and functional outcomes after TAA.

Improvements in biomechanical designs and surgical techniques have resulted in better satisfaction and clinical outcomes following the TAA. Relief of pain, enhancement of function, and improved quality of life enable patients to engage more actively in physical exercise and sports [21]. The targeted rehabilitation process to enhance abilities for daily living and sports activities after TAA may be useful for better clinical outcomes and patients’ satisfaction. Engagement in sports activity may play a key role in overall health and constitute an important aspect of social interaction. Valderrabano et al. found a significant postoperative increase in sports participation following TAA, as well as a significant association between sports activity levels and the American Orthopaedic Foot and Ankle Society hindfoot score [16]. The clinical scoring questionnaires (FAOS and FAAM) used in this study included assessments of ability for sports activities, enabling the evaluation of functional ability that requires greater physical activity than daily living.

Because proprioceptive information from joints and periarticular soft tissue is essential for keeping a stable posture and normal gait [22,23], bone resection and soft tissue manipulation by the total joint replacement may inevitably result in changes in proprioception and postural control [24,25]. In regard to the total hip or knee arthroplasty, reestablishment of joint space and soft tissue tension and improvement of chronic inflammation were reported to enhance mechanoreceptors in both capsuloligamentous and musculotendinous structures and to improve the proprioception [26]. However, the effects of TAA on the entire proprioception including postural control ability remain still unclear. Butler et al. reported that just 9% of TAA patients were able to successfully complete a single-leg stance test one year postoperatively, compared with 63% of total hip patients and 69% of total knee patients who passed the same balance assessment [24].

Conti et al. reported that TAA did not create a proprioceptive deficit compared to the contralateral ankle [11]. Lee et al. have also reported that the presence of deficits in proprioception despite capsulectomy during TAA was not demonstrated [14]. They found that patients who underwent TAA exhibited more pronounced dynamic postural imbalance than matched healthy controls of similar age and sex [14]. Zeininger et al. reported that ankle OA patients showed minimal side-to-side asymmetry in the center of pressure (COP) pathway before surgery, and this asymmetry between the operated and unaffected ankles was reduced to a relatively normal path after TAA [7]. In regard to the ligament balancing during TAA, Doty et al. reported that deltoid ligament release for varus deformity did not appear to accelerate medial ankle instability or collapse of the longitudinal arch of the foot [8]. Although both static and dynamic postural control abilities showed a trend toward better stability index after TAA in the current study, no statistically significant differences were found when compared to preoperative evaluation.

Another question is whether postoperative static and dynamic postural control ability can be improved through continuous proprioceptive-oriented rehabilitation after TAA. Although an evaluation of postural control ability may be useful as an indicator of the functional restoration for patients with a rehabilitation program after TAA, objective and quantitative evaluation methods have yet to be adequately established. In comparison to analysis for dynamic balance, the measurement of static balance is likely to show statistically non-significant differences in the ankle joints with OA compared to the unaffected side or healthy control group [27]. This report from Scaturro et al. seems to be in line with the results in the current study. FAOS demonstrated no significant correlation with static postural control, but showed a low linear association with dynamic postural control. Similarly, FAAM scores did not correlate significantly with static postural control, but demonstrated a moderate linear association with dynamic postural control. In regard to the minimal clinically important differences (MCIDs) for FAOS and FAAM, while FAAM generally presents 8 points on the activity for daily living (ADL) subscale and 9 points on the sports subscale as MCIDs, FAOS has not presented the established representative MCID values consistently. Therefore, FAAM allows for the relatively clear application of criteria for each subscale, whereas FAOS requires greater caution in interpretation because the scope is often broad or separate calculation values are frequently needed depending on the research.

Age is known to be associated with impaired proprioception, muscle weakness, insufficient stamina, and deteriorated neuromuscular control [28,29]. Joint damage in OA patients has been reported to be associated with a proprioceptive deficiency that causes less accuracy in postural control and more limited movement to avoid losing balance [14,30,31]. In particular, ankle pain aggravated by weight-bearing during single-leg stance in patients with severe ankle arthritis can be a significant confounding factor in the assessment of postural control ability. The contralateral side is also highly likely to have an existing age-related proprioceptive deficit, so it may not be suitable as a control group for comparative study. Age and BMI were reported to be the most significant factors influencing dynamic postural balance in both RA patients and healthy controls [13]. In a previous study using the Biodex Stability System, Aydoğ et al. reported that the marked differences in dynamic postural balance were found between RA and healthy subjects [13]. In addition, a significant correlation between functional disability (evaluated with the Health Assessment Questionnaire) and postural balance was detected.

Balance enhancement training for the elderly or patients with degenerative arthritis has tended not to receive much attention until now. Appropriate balance control ability and proprioception in elderly and OA patients may be critical to better physical functioning and prevention of falling. Suomi and Kojeca reported that aquatic exercise treatment for increasing postural stability reduced significantly postural sway in women with lower extremity arthritis [32]. Truque-Díaz et al. demonstrated that bilateral hemophilic ankle arthropathy was associated with poorer postural stability and balance in the absence of visual feedback compared with healthy controls [31]. They have suggested that repeated hemarthrosis in the same joint leads to the functional deterioration of the proprioceptive mechanical receptors and cartilage degeneration. To date, standardized rehabilitation protocols targeting postural control recovery after TAA have not yet been established. As an at-home exercise, we think that TAA patients need to have continuous balance training such as static tandem standing (from eyes open to closed), single-leg standing on an unstable surface (wobble board), and single-leg standing with ball toss.

This study has some limitations. First, this study included only one kind of TAA design and all surgeries were performed by one surgeon in one institute. The variability among the TAA prosthesis systems and details of surgical procedures may lead to heterogeneous results, and limit the general applicability of research findings. Second, the existing age-related degeneration may negatively affect exact evaluations for proprioceptive changes following the ankle joint replacement. Age-related decline in lower limb muscle strength, decreased neuromuscular control, and concomitant degenerative changes in other joints may all contribute to a decrease in postural control ability. Third, this study included no control group matched by age and sex. It is controversial as to whether patients with contralateral ankles in end-stage arthritis are an ideal control group for exact side-to-side comparison of postural control ability. Because there is insufficient information on proprioception and postural control ability in normal elderly populations, further studies need to consider recruitment of an age- and sex-matched healthy control group. Fourth, there was a lack of subgroup or multivariable analysis on important confounding variables such as age, BMI, osteoporosis, muscle weakness, and severity of preoperative deformity.

## 5. Conclusions

TAA failed to bring a statistically significant improvement in postural control ability. Dynamic postural control ability after TAA was found to have a significant correlation with patient-reported clinical outcomes. To enhance postoperative clinical outcomes, including abilities for daily living and sports activities, biomechanical assessment and targeted rehabilitation strategies to improve postural control ability may be helpful.

## Figures and Tables

**Table 1:** Patients’ demographic and clinical characteristics.

Sex (no. [%])	
Female	18 (56.2)
Male	14 (43.8)
Age at surgery (year) *	63.2 ± 9.1
Body mass index (kg/m^2^) *	25.6 ± 2.6
Bone mineral density (g/cm^2^) *	1.13 ± 0.5
Smoking (no. [%])	
Never	15 (46.9)
Former	12 (37.5)
Smoker	5 (15.6)
Diabetes (no. [%])	5 (15.6)
Hypertension (no. [%])	9 (28.1)
Osteoporosis (T-score ≤ −2.5)	3 (9.4)
Length of follow-up (month) *	51.2 ± 15.8

* Data are presented as mean ± standard deviation (SD).

**Table 2:** Clinical outcomes assessed with the Foot and Ankle Outcome Score, the Foot and Ankle Ability Measure.

Subscales	Preoperative	PO 6 Months	PO 1 Year	Final F/U	*p*-Value ^†^
FAOS *					
Pain	29.8 ± 14.1	74.7 ± 13.3	82.9 ± 10.4	86.1 ± 8.2	<0.001
Symptoms	35.6 ± 16.5	73.5 ± 12.9	84.2 ± 9.7	84.5 ± 8.7	<0.001
Activity of daily living	37.5 ± 17.2	79.2 ± 12.6	85.1 ± 9.9	88.6 ± 7.5	<0.001
Sports	24.3 ± 13.4	46.6 ± 16.5	63.7 ± 13.4	68.2 ± 11.3	<0.001
Quality of life	35.3 ± 16.8	75.4 ± 13.1	86.3 ± 10.2	84.1 ± 9.6	<0.001
**Total FAOS**	32.5 ± 14.8	69.9 ± 14.2	80.4 ± 11.8	82.3 ± 10.4	<0.001
FAAM *					
Daily activity	39.8 ± 18.7	79.5 ± 13.1	87.1 ± 9.3	90.4 ± 7.8	<0.001
Sports activity	28.4 ± 15.5	48.9 ± 17.6	67.2 ± 13.1	76.8 ± 10.9	<0.001
**Total FAAM score**	34.1 ± 15.4	64.2 ± 15.5	77.2 ± 12.5	83.6 ± 10.7	<0.001

Abbreviation: PO, postoperative; F/U, follow-up. * Scores were converted to a 100-point scale and are reported as mean ± standard deviation. ^†^ Comparison between preoperative and final follow-up.

**Table 3:** Static postural control ability evaluated with the Biodex posturography.

Biodex Posturography	Preoperative	PO 6 Months	PO 1 Year	Final F/U	*p*-Value ^†^
A-P stability index *	1.38 ± 0.65	1.41 ± 0.73	1.28 ± 0.62	1.25 ± 0.64	0.403
M-L stability index *	1.02 ± 0.54	1.23 ± 0.62	0.96 ± 0.49	0.92 ± 0.51	0.659
Overall stability index *	1.63 ± 0.76	1.88 ± 0.87	1.59 ± 0.71	1.52 ± 0.69	0.551

Abbreviation: PO, postoperative; F/U, followup; A-P, anterior–posterior; M-L, medial–lateral. * Values are reported as mean ± standard deviation. ^†^ Comparison between preoperative and final follow-up.

**Table 4:** Dynamic postural control ability evaluated with the Biodex posturography.

Biodex Posturography	Preoperative	PO 6 Months	PO 1 Year	Final F/U	*p*-Value ^†^
A-P stability index *	2.05 ± 0.98	2.13 ± 1.12	2.15 ± 1.09	1.91 ± 0.92	0.515
M-L stability index *	1.69 ± 0.79	1.72 ± 0.81	1.76 ± 0.82	1.58 ± 0.73	0.629
Overall stability index *	2.74 ± 1.28	2.78 ± 1.32	2.81 ± 1.33	2.56 ± 1.14	0.348

Abbreviation: PO, postoperative; F/U, follow-up; A-P, anterior–posterior; M-L, medial–lateral. * Values are reported as mean ± standard deviation. ^†^ Comparison between preoperative and final follow-up.

**Table 5:** Correlation between postural control ability and clinical outcome measures.

Spearman’s Correlation Coefficients	Posturography	
	OSI (Static Mode)	OSI (Dynamic Mode)
Clinical outcome measures		
FAOS (at final followup)	r = −0.11	r = −0.35
*p*-value ^†^	0.425	0.021
FAAM (at final followup)	r = −0.26	r = −0.57
*p*-value ^‡^	0.103	<0.001

Abbreviation: OSI, Overall stability index; FAOS, Foot and Ankle Outcome Score; FAAM, Foot and Ankle Ability Measure. ^†^ Comparison between FAOS and postural control ability (OSI). ^‡^ Comparison between FAAM and postural control ability (OSI).

## Data Availability

The data presented in this study are available in the article.

## Abbreviations

The following abbreviations are used in this manuscript:

TAA	Total Ankle Arthroplasty
MBP	Modified-Broström procedure
RTS	Return To Sports
FAOS	Foot and Ankle Outcome Score
FAAM	Foot and Ankle Ability Measure
